# Radiographic and α-fetoprotein response predict pathologic complete response to immunotherapy plus a TKI in hepatocellular carcinoma: a multicenter study

**DOI:** 10.1186/s12885-023-10898-z

**Published:** 2023-05-09

**Authors:** Cheng Huang, Xiao-Dong Zhu, Ying-Hao Shen, Bin Xu, Dong Wu, Yuan Ji, Ling-Li Chen, Tian-Qiang Song, Wei Zhang, Zhi-Ming Zeng, Hua-Sheng Huang, Kui Wang, Lan-Qing Huang, Yong-Jun Chen, Yu-Chen Yang, Le-Du Zhou, Guo Long, Hai-Tao Zhao, Yun-Chao Wang, Ning-Ling Ge, Yi Chen, Chang-Jun Tan, Jian Zhou, Jia Fan, Hui-Chuan Sun

**Affiliations:** 1 Department of Liver Surgery and Transplantation, Liver Cancer Institute and Zhongshan Hospital, Fudan University, 180 Fenglin Road, Shanghai, 200032 China; 2 Department of Radiology, Zhongshan Hospital, Fudan University, Shanghai, China; 3 Department of Pathology, Zhongshan Hospital, Fudan University, Shanghai, China; 4 Department of Hepatobiliary, Oncology Key Laboratory of Cancer Prevention and Therapy, National Clinical Research Center of Cancer, Tianjin Medical University Cancer Institute and Hospital, Tianjin, China; 5 Department of Medical Oncology, The First Affiliated Hospital of Guangxi Medical University, Nanning, Guangxi Zhuang Autonomous Region China; 6 Department of Hepatic Surgery II, Eastern Hepatobiliary Surgery Hospital, Navy Medical University, Shanghai, China; 7 Department of Hepatobiliary Surgery, Ruijin Hospital, Shanghai Jiao Tong University School of Medicine, Shanghai, China; 8 Department of General Surgery, Xiangya Hospital, Central South University, Changsha, China; 9 Department of Liver Surgery, Peking Union Medical College Hospital, Chinese Academy of Medical Sciences and Peking Union Medical College, Beijing, China; 10 Department of Hepatic Oncology, Liver Cancer Institute and Zhongshan Hospital, Fudan University, Shanghai, China

**Keywords:** Tyrosine kinase inhibitor, Immunotherapy, Hepatocellular carcinoma, Conversion therapy, Pathologic complete response

## Abstract

**Background:**

Pathologic complete response (pCR) following preoperative systemic therapy is associated with improved outcomes after subsequent liver transplant/resection in hepatocellular carcinoma (HCC). However, the relationship between radiographic and histopathological response remains unclear.

**Methods:**

We retrospectively examined patients with initially unresectable HCC who received tyrosine kinase inhibitor (TKI) plus anti–programmed death 1 (PD-1) therapy before undergoing liver resection between March 2019 and September 2021 across 7 hospitals in China. Radiographic response was evaluated using mRECIST. A pCR was defined as no viable tumor cells in resected samples.

**Results:**

We included 35 eligible patients, of whom 15 (42.9%) achieved pCR after systemic therapy. After a median follow-up of 13.2 months, tumors recurred in 8 non-pCR and 1 pCR patient. Before resection, there were 6 complete responses, 24 partial responses, 4 stable disease cases, and 1 progressive disease case, per mRECIST. Predicting pCR by radiographic response yielded an area under the receiver operating characteristic curve (AUC) of 0.727 (95% CI: 0.558–0.902), with an optimal cutoff value of 80% reduction in the enhanced area in MRI (called major radiographic response), which had a 66.7% sensitivity, 85.0% specificity, and a 77.1% diagnostic accuracy. When radiographic response was combined with α-fetoprotein response, the AUC was 0.926 (95% CI: 0.785–0.999); the optimal cutoff value was 0.446, which had a 91.7% sensitivity, 84.6%, specificity, and an 88.0% diagnostic accuracy.

**Conclusions:**

In patients with unresectable HCC receiving combined TKI/anti–PD 1 therapy, major radiographic response alone or combined with α-fetoprotein response may predict pCR.

## Background

The majority of patients diagnosed with hepatocellular carcinoma (HCC) are not eligible for potentially curative treatment with surgical resection or ablation due to being in an advanced stage of disease, or other factors including comorbidities and underlying liver disease [1, 2]. Patients with advanced, unresectable HCC at diagnosis are usually treated with locoregional therapies or systemic therapy instead [3]. From 2007 to 2018, sorafenib was the only systemic treatment available for HCC, but in recent years new first-line treatments have been approved, including the tyrosine kinase inhibitor (TKI) lenvatinib [4] as well as combination treatment with the anti–programmed death-ligand 1 antibody atezolizumab plus the antiangiogenic agent bevacizumab [5]. Both lenvatinib and atezolizumab plus bevacizumab resulted in higher objective response rates (ORRs) versus sorafenib in patients with advanced HCC, as reported in the Phase III REFLECT trial (24.1% vs. 9.2%) per the modified Response Evaluation Criteria in Solid Tumors (mRECIST) [6], and the IMbrave150 trial (35.4% vs. 13.9%) per mRECIST, respectively [4, 7]. Furthermore, early-stage trials of combination therapy with lenvatinib plus the anti–programmed death 1 (PD-1) antibodies pembrolizumab or nivolumab have similarly reported high ORRs of 40.3% and 54.2%, respectively, in patients with HCC [8, 9].

The high ORRs achieved with novel systemic therapies in advanced HCC have revived interest in the concept of conversion therapy. Conversion therapy aims to use systemic treatment to downstage patients with initially unresectable and advanced HCC, to provide an opportunity for these patients to undergo curative resection. This approach offers the chance of long-term survival to those who achieve a good response to systemic therapy. Although conversion therapy remains an investigative treatment strategy, early findings are promising, with multiple reports showing this approach is feasible and safe [10–12]. In particular, retrospective analyses have shown that combination treatment with TKIs and anti–PD-1 antibodies have reduced tumor burden sufficiently in a proportion of patients with advanced HCC, enabling them to undergo R0 resection [13–15]. A study by Ho et al. demonstrated that patients’ postoperative survival rates were associated with the magnitude of pathological response to pre-operative systemic therapy [14]. Therefore, further research is needed to determine the optimal method of evaluating treatment response to systemic therapy as part of a conversion therapy strategy, as this will aid accurate selection of patients suitable for surgical resection. Indeed, achieving a pathologic complete response (pCR) to locoregional therapy before liver transplantation has been identified as an independent predictor of longer overall survival [16].

Most international guidelines recommend that response to locoregional and systemic treatment in patients with advanced HCC should be assessed using both RECIST 1.1 and mRECIST [6]. These evaluation criteria were developed to obtain objective measurements of tumor response from radiographic observations [17]. While RECIST is based solely on change in tumor size during treatment, mRECIST was developed as an adaptation of RECIST to assess changes in response to locoregional and targeted therapies in HCC and is based on change in viable tumor tissue (a marker of treatment-induced tumor necrosis) by contrast-enhanced imaging [17]. Despite the widespread use of radiography for assessing tumor response in clinical practice and clinical trials, histopathological response, as evaluated using resected tumor tissue, remains the gold standard. Furthermore, in our previous study, we found that α-fetoprotein (AFP) response within 1 week after surgical resection can be used to evaluate the oncologic effect of hepatectomy for HCC and was an independent predictive factor of overall survival and recurrence-free survival [18].

Given this background, the purpose of this study was to explore the relationship between radiographic response alone using the mRECIST measurement method or combined with AFP response and pCR, in patients with HCC receiving systemic therapy with a combination of a TKI and an anti–PD-1 antibody followed by surgical resection.

## Methods

### Study design and patients

This retrospective analysis included consecutive patients with an initial diagnosis of unresectable HCC who underwent resection after receiving combined TKI and anti–PD-1 antibody treatment between March 2019 and September 2021 at 7 hospitals in China. These patients were unresectable mainly because of tumor characteristics (e.g., too large tumors with insufficient future liver remnant volume, Barcelona Clinic Liver Cancer stage B or C disease). Of them, patients from Zhongshan Hospital, Fudan University were retrospectively recruited from an ongoing observational, prospective cohort study (NCT04639284). Eligibility for liver resection after combination therapy was assessed according to previously published criteria [13]. The diagnosis of HCC was based on tissue histology or clinical manifestations according to the American Association for the Study of Liver Diseases criteria [19]. Eligible patients were also required to undergo a pretreatment contrast-enhanced magnetic resonance imaging (MRI) within 2 weeks before initiating combination therapy, have tumor response assessments every 2 months (± 2 weeks) via contrast-enhanced MRI according to mRECIST, and have at least one tumor response assessment after initiating combination therapy.

Patients who received preoperative locoregional therapy (e.g., transcatheter arterial chemoembolization, hepatic artery infusion chemotherapy, portal vein embolization) or another type of systemic treatment (e.g., chemotherapy) were not eligible for this study. The other exclusion criteria were: incomplete clinicopathologic data; a history of cancer other than HCC; intrahepatic tumor lesions that could not be measured in magnetic resonance images; and additional anti-tumor treatment after the initiation of combination therapy and before conversion surgery.

All patients received lenvatinib (8 mg/day regardless of body weight; Eisai, Inc., Japan) or apatinib (250 mg/day Hengrui Medicine, China) [20, 21] plus an anti–PD-1 antibody. Anti–PD-1 antibodies were intravenously administered at the following dose regimens: nivolumab (Bristol-Myers Squibb, USA) 3 mg/kg or camrelizumab (Hengrui Medicine, China) 200 mg [22] every 2 weeks, or pembrolizumab (MSD, USA) 200 mg, sintilimab (Innovent Biologics, China) 200 mg [23], or toripalimab (Junshi Bioscience, China) 240 mg [24] every 3 weeks. All patients with active hepatitis B virus (HBV) infection received concomitant antiviral therapy. All patients received ≥ 3 infusions of anti–PD-1 antibodies and underwent regular monitoring for efficacy and safety assessment, including repeat safety evaluations 2–3 days prior to each anti–PD-1 antibody treatment cycle.

The study was conducted in accordance with the Declaration of Helsinki and written informed consent was obtained from all patients before inclusion in the study. The study protocol was approved by the Zhongshan Hospital Research Ethics Committee (Approval Numbers: B2020-177R).

### Measurements

Prior to treatment, all patients underwent a baseline evaluation that included liver, renal, thyroid, adrenal, and cardiac function tests; complete blood count analysis; and testing for hepatitis B surface antigen (HBsAg), HBV DNA, and AFP. AFP response was defined as a change from a positive status at baseline to a negative status before resection.

Tumor responses were evaluated using abdominal contrast-enhanced MRI and chest serial computed tomography every 2 months (± 2 weeks). The radiographic response was defined as the percentage of reduction in tumor size in MRI using the mRECIST measurement method (i.e., the percentage of reduction in tumor size of enhanced tumor area in MRI), and its evaluation was performed by independent imaging review groups [17]. Hepatic lesions (≥ 1 cm) were selected for repeated assessment (target lesions) if they could be accurately measured in at least one dimension, with the final selection of target lesions based on size (those with the longest diameter) and suitability for accurate repeated measurements. The ORR was defined as the percentage of patients with a complete or partial response (CR or PR).

### Surgical resection

The surgical resection procedure has been described in our previous study. Briefly, Intraoperative ultrasonography was used to visualize the location of the tumor and its relationship with major vascular structures, as well as to detect satellite nodules. Parenchyma transection was conducted by alternating use of an ultrasonic dissector and the clamp-crushing technique. Complete hemostasis was achieved by ligation or electrocoagulation. The Pringle maneuver or hepatic vein occlusion was used to control bleeding from inflow or outflow vessels if necessary [25].

### Pathological evaluation

Pathological analysis was conducted on tissue specimens obtained during resection surgery to confirm the diagnosis of HCC in all patients. Macroscopic evaluation of specimens was used to calculate the overall size and number of tumors, presence of satellite nodules, and presence of necrosis. Microscopic analysis was used to confirm tumor differentiation, fatty changes, septum formation, capsule invasion, microvascular invasion, surgical margin invasion, and cirrhosis of the nontumor liver portion. Tumor size was determined as the largest tumor diameter, and tumor cell differentiation was evaluated using the Edmondson-Steiner grading system (grades I to IV).

Histopathological response was evaluated by calculating the residual viable tumor (RVT; RVT area/total tumor bed surface area under 100 × microscopy) by two pathologists (YJ and LLC). A pCR was defined as the detection of no RVT cells on hematoxylin and eosin–stained slide sections among all resected primary tumor(s), tumor thrombosis, and metastatic lesions. If no viable tumor cells were identified in the initially evaluated tissue samples, additional samples were evaluated for confirmation. The number of additional samples was determined based on the size of the tumor bed and the capacity of the pathology department. If there was disagreement between the histological findings and imaging evaluation, the possibility of sampling position misalignment was considered. A re-evaluation of the gross specimen to establish a better agreement between imaging and pathological sample location in the gross tumor specimen was conducted. If new lesions were found during this process, they were resampled.

### Statistical analysis

A sample size of at least 26 patients (13 patients with a pCR and 13 patients without a pCR) was required based on the following assumptions: a power of 0.8, two-sided α of 0.05, alternative hypothesis of an area under the receiver operating characteristic (ROC) curve (AUC) of 0.8 compared with the null hypothesis of an AUC of 0.5, and an expected pCR rate of around 50% based on a previous report [13]. Sample size was calculated using PASS 2021 (NCSS, LLC, Kaysville, UT, USA).

Statistical analyses were performed using R software (version 4.1.2). Results were summarized using descriptive statistics: continuous variables were summarized as mean (standard deviation) or median (interquartile range) unless otherwise specified, and binary variables were summarized as n (%). The ORR and associated 95% confidence intervals (CI) were calculated using the Clopper-Pearson method (https://epitools.ausvet.com.au/). Time to recurrence (TTR) was defined as the interval between hepatic resection and recurrence. TTR was estimated using the Kaplan–Meier method and the log-rank test was used to compare survival for patients who did and did not achieve a pCR. Logistic regression and ROC curve analyses were used to evaluate the relationships between radiographic tumor response assessed by mRECIST and pCR or between radiographic tumor response combined with AFP response and pCR. To perform an internal validation, bootstrap resampling (*n* = 1000) was used to calculate an AUC with 95% CI and compare the difference between two ROC curves. The optimal cutoff value was determined using ROC analysis by maximizing the Youden index (sensitivity plus specificity minus 1). The Delong test was used to compare differences between AUCs. The Pearson coefficient was calculated to assess the correlation between radiographic response and degree of tumor necrosis assessed by pathology. All statistical tests were 2-sided and *P* < 0.05 was considered statistically significant.

## Results

### Baseline patient characteristics

**Fig. 1:**
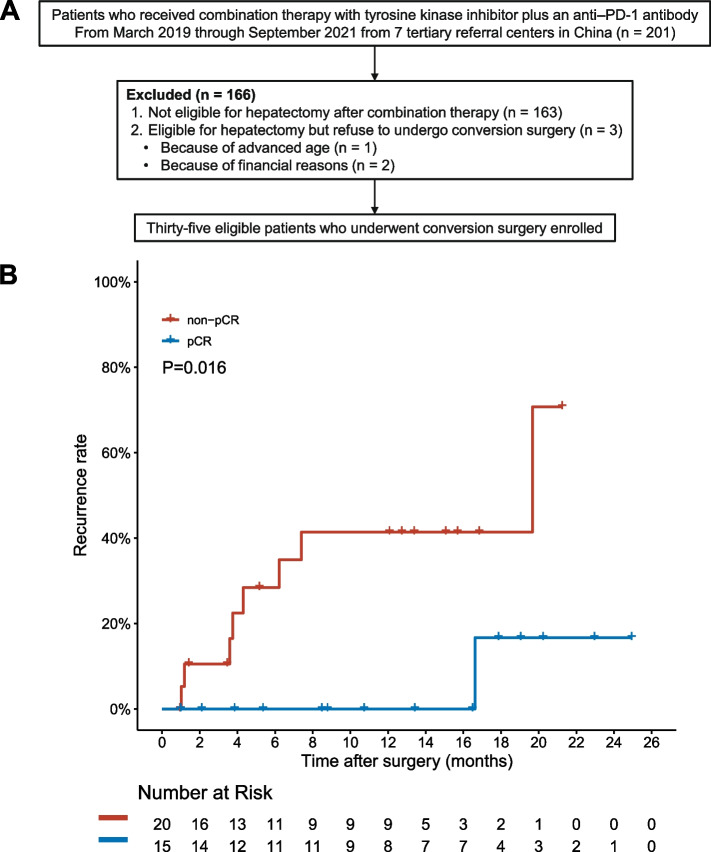
Flowchart of patient enrollment and time to recurrence. **A** Flowchart of patient enrollment. **B** Time to recurrence in patients who did and did not achieve a pCR following systemic therapy after surgical resection (*P* = 0.016). pCR, pathologic complete response; PD-1, programmed death 1

Of 201 patients included, 35 patients were eligible for this study (Fig. 1A, Table 1), with a conversion rate of 17.4% (35/201), which was comparable with our previous study [13]. All patients achieved R0 resection after systemic therapy. Of them, 15 patients achieved a pCR after systemic therapy. At baseline, the majority of patients were male (94.3%), HBsAg positive (82.9%), had Child–Pugh class A liver function (100%), and had Barcelona Clinic Liver Cancer stage C disease (66%). Patients received lenvatinib (*n* = 31) or apatinib (*n* = 4) plus an anti–PD-1 antibody (pembrolizumab [*n* = 5], sintilimab [*n* = 11], camrelizumab [*n* = 15], treprizumab [*n* = 2], tislelizumab [*n* = 1], or nivolumab [*n* = 1]).

**Table 1:** Patient demographics and baseline characteristics

Variables	All patients (*N* = 35)
Age, mean ± SD, years	56.4 ± 9.13
Sex, n (%)	
Female	2 (6)
Male	33 (94)
HBsAg, n (%)	
Negative	6 (17)
Positive	29 (83)
Child–Pugh grade, n (%)	
A	35 (100)
AFP at baseline, n (%)	
≤ 400 ng/mL	13 (37)
> 400 ng/mL	22 (63)
AFP at baseline, n (%)	
≤ ULN	10 (29)
> ULN	25 (71)
AFP before surgery, n (%)	
≤ ULN	15 (43)
> ULN	20 (57)
BCLC stage, n (%)	
A	3 (9)
B	9 (26)
C	23 (66)
CNLC stage, n (%)	
Ib	3 (9)
IIa	1 (3)
IIb	8 (23)
IIIa	13 (37)
IIIb	10 (29)
Macrovascular invasion, n (%)	
No	21 (60)
Yes	14 (40)
Extrahepatic disease, n (%)	
No	25 (71)
Yes	10 (29)
Location of extrahepatic disease, n (%)	
Abdominal cavity	1 (3)
Adrenal gland	1 (3)
Bone^a^	3 (9)
Lung	2 (6)
Lymph node^a^	4 (11)
Anti–PD-1 antibody treatment cycle before surgery, median (IQR)	5 (3–8)
Time to surgery, median (IQR), months	4.4 (2.73–6.57)
Radiographic response^b^, median (IQR)	65% (48–87)

*AFP* α-fetoprotein, *BCLC* Barcelona Clinic Liver Cancer, *CNLC* China National Liver Cancer, *ECOG* Eastern Co-operative Oncology Group, *HBsAg* Hepatitis B surface antigen, *IQR* Interquartile range, *mRECIST* Modified Response Evaluation Criteria in Solid Tumors, *PD-1* Programmed death 1, *SD* Standard deviation, *ULN* The upper limit of normal at each patient’s respective hospital

^a^One patient had both hilar lymph node and lumbar vertebra metastases

^b^Using the mRECIST measurement method

A higher proportion of the patients who achieved a pCR had macrovascular invasion compared with those who did not achieve a pCR (60% vs. 25%, *P* = 0.081, Table 2), but not statistically significant. Among baseline clinicopathological parameters, negative AFP before surgery and AFP response were associated with pCR (*P* = 0.043 and *P* = 0.009, respectively, Table 2), indicating that whether a pCR can be achieved may be mainly determined by the biology of tumors. The median time from initiation of systemic therapy to surgical resection in patients who achieved a pCR and those who did not was 3.73 months and 4.42 months, respectively.

**Table 2:** Baseline characteristics split by pathological response

Variables	Non-pCR (*n* = 20)	pCR (*n* = 15)	*P* value
Age, mean ± SD, years	56.6 ± 9.85	56.13 ± 8.4	0.881
Sex, n (%)			0.496
Female	2 (10)	0 (0)	
Male	18 (90)	15 (100)	
HBsAg, n (%)			0.207
Negative	5 (25)	1 (7)	
Positive	15 (75)	14 (93)	
Child–Pugh grade, n (%)			-
A	20 (100)	15 (100)	
AFP at baseline, n (%)			0.449
≤ 400 ng/mL	9 (45)	4 (27)	
> 400 ng/mL	11 (55)	11 (73)	
AFP at baseline, n (%)			0.458
≤ ULN	7 (35)	3 (20)	
> ULN	13 (65)	12 (80)	
AFP before surgery, n (%)			0.043
≤ ULN	8 (40)	12 (80)	
> ULN	12 (60)	3 (20)	
AFP response^a^, n (%)			0.009
No	11 (85)	3 (25)	
Yes	2 (15)	9 (75)	
BCLC stage, n (%)			0.867
A	2 (10)	1 (7)	
B	6 (30)	3 (20)	
C	12 (60)	11 (73)	
CNLC stage, n (%)			0.525
Ib	2 (10)	1 (7)	
IIa	1 (5)	0 (0)	
IIb	5 (25)	3 (20)	
IIIa	5 (25)	8 (53)	
IIIb	7 (35)	3 (20)	
Macrovascular invasion, n (%)			0.081
No	15 (75)	6 (40)	
Yes	5 (25)	9 (60)	
Extrahepatic disease, n (%)			0.458
No	13 (65)	12 (80)	
Yes	7 (35)	3 (20)	
Location of extrahepatic disease, n (%)			0.320
Abdominal cavity	0 (0)	1 (7)	
Adrenal gland	1 (5)	0 (0)	
Bone^b^	3 (15)	0 (0)	
Lung	1 (5)	1 (7)	
Lymph node^b^	3 (15)	1 (7)	
Anti–PD-1 antibody treatment cycle before surgery, median (IQR)	5 (3.75–7.25	4 (3, 9)	0.867
Time to surgery, median (IQR), months	4.42 (3.04–5.68)	3.73 (2.6–7.48)	0.907
Radiographic response^c^, median (IQR)	59% (38–77)	87% (58–95)	0.023

*AFP* α-fetoprotein, *BCLC* Barcelona Clinic Liver Cancer, *CNLC* China National Liver Cancer, *ECOG* Eastern Co-operative Oncology Group, *HBsAg* Hepatitis B surface antigen, *IQR* Interquartile range, *mRECIST* Modified Response Evaluation Criteria in Solid Tumors, *pCR* Pathologic complete response, *SD* Standard deviation, *ULN* The upper limit of normal at each patient’s respective hospital

^a^AFP response was defined as a change from a positive status at baseline to a negative status before resection. Of all 35 patients, 25 patients had positive AFP at baseline

^b^One patient had both hilar lymph node and lumbar vertebra metastases

^c^Using the mRECIST measurement method

### The association between pCR and postresection recurrence

After a median follow-up time of 13.2 months (range: 1.0–27.6 months) following hepatectomy, 8/20 (40.0%) of the patients who had not achieved a pCR experienced tumor recurrence while 1/15 (6.7%) of the patients who had achieved pCR experienced tumor recurrence. Moreover, TTR was significantly longer for the patient who had achieved a pCR (hazard ratio: 0.116, 95% CI: 0.014–0.942, *P* = 0.016; Fig. 1B).

### Relationship between pathological and radiographic responses

**Fig. 2:**
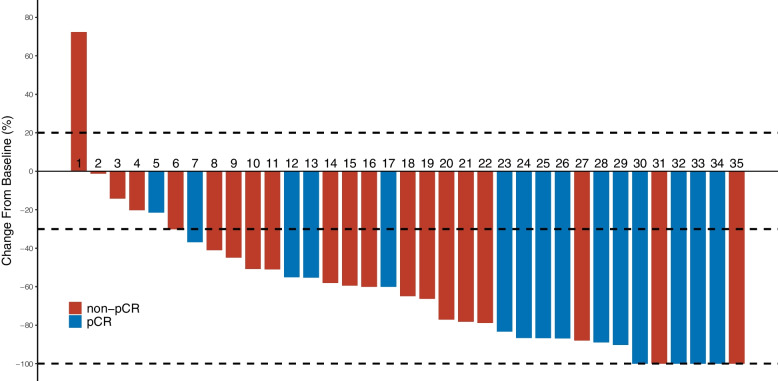
Radiographic response assessment for patients with unresectable HCC receiving combined treatment with a tyrosine kinase inhibitor and an anti–PD-1 antibody as assessed by mRECIST. Each bar represents 1 patient. The blue bars indicate patients who achieved a pCR, and the red bars indicate those who did not achieve a pCR after systemic therapy. The intrahepatic tumor of Patient 1 was evaluated as a PR; however, a new metastatic lesion was found in the right adrenal gland during systemic therapy. HCC, hepatocellular carcinoma; mRECIST, modified Response Evaluation Criteria in Solid Tumors; pCR, pathologic complete response; PD-1, programmed death 1

**Fig. 3:**
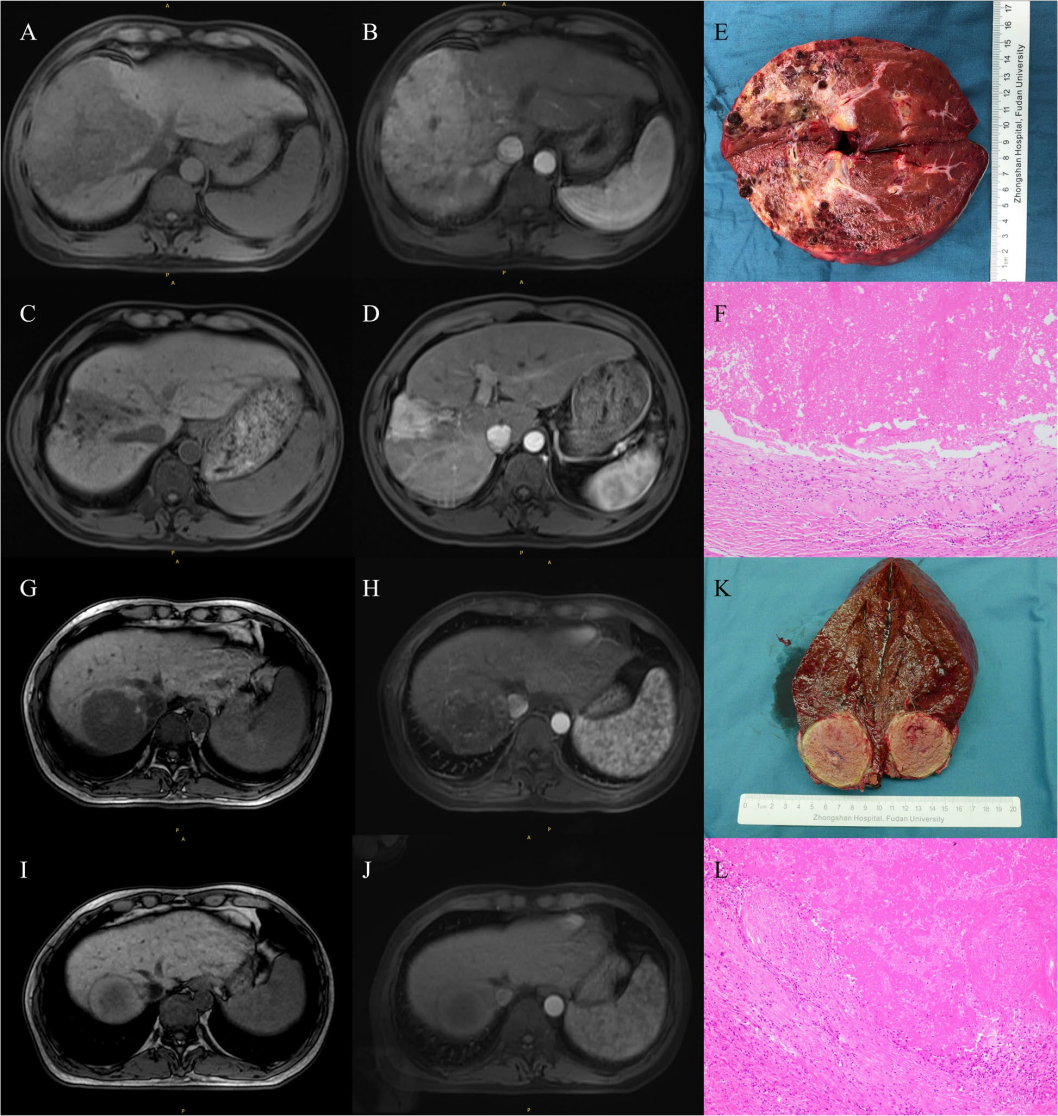
Representative images comparing radiographic and pathological response evaluations. **A-F** Representative figures collected from patient case 13, who was evaluated as radiographic PR per mRECIST and pCR. **A, B** MR images before combination therapy with a tyrosine kinase inhibitor and an anti–PD-1 antibody and (**C, D**) MR images after systemic therapy, but before surgery. **E** The resected tumor tissues and (**F**) the hematoxylin and eosin–stained slide section (× 100). **G-L** Representative figures collected from patient case 34, who was evaluated as radiographic CR per mRECIST and pCR. **G, H** MR images before combination therapy with a tyrosine kinase inhibitor and an anti–PD-1 antibody and (**I, J**) MR images after systemic therapy, but before surgery. **K** The resected tumor tissues and (L) the hematoxylin and eosin–stained slide section (× 100). CR, complete response; MR, magnetic resonance; mRECIST, modified Response Evaluation Criteria in Solid Tumors; pCR, pathologic complete response; PR, partial response; PD-1, programmed death 1

As evaluated by MRI per mRECIST before surgical resection, of the 35 study patients, 6 achieved a CR, 24 achieved a PR, 4 achieved stable disease (SD), and 1 had progressive disease (PD; the intrahepatic tumor was evaluated as a PR; however, a new metastatic lesion was found in the right adrenal gland during systemic therapy), resulting in an ORR of 85.7% (Fig. 2). Fewer patients achieved a radiographic CR (*n* = 6) than those who achieved a pCR (*n* = 15); 10 patients who achieved a radiographic PR and 1 patient who achieved radiographic SD also achieved a pCR, suggesting that a radiographic CR is not necessary for a patient to have achieved a pCR. A comparison between radiographic and pathological response evaluation is presented in Fig. 3.

### Radiographic response as a predictor of pCR

**Fig. 4:**
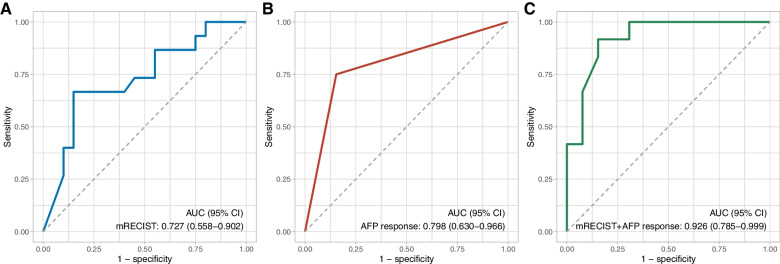
Receiver operating characteristic curves to predict pCR. Receiver operating characteristic curves for the ability of (**A**) the radiographic response (using the mRECIST measurement method), **B** AFP response, and **C** the radiographic response combined with AFP response to predict pCR, respectively. **A** included all 35 patients; **B** and **C** only included 25 patients who had positive AFP at baseline. AFP, α-fetoprotein; AUC, area under the receiver operating characteristic curve; mRECIST, modified Response Evaluation Criteria in Solid Tumors; pCR, pathologic complete response

As shown in Table 2, radiographic response was associated with pCR. To test the ability of radiographic response to predict pCR, we examined the radiographic AUC, which we found to be 0.727 (95% CI: 0.558–0.902, Fig. 4A). The optimal cutoff value for predicting pCR was an 80% reduction in the enhanced tumor area in MRI, which was defined as a major radiographic response (MRR). Using this criterion, we observed a sensitivity of 66.7%, specificity of 85.0%, positive predictive value (PPV) of 76.9%, negative predictive value (NPV) of 77.3%, and a diagnostic accuracy of 77.1% for predicting pCR.

### Combination of radiographic response and AFP response for predicting pCR

Of the 35 patients, 25 (71.4%) were AFP positive (higher than upper limit of normal at each patient’s respective hospital) at baseline. In 25 patients who had positive AFP at baseline, AFP response resulted in an AUC of 0.798 (95% CI: 0.630–0.966, Fig. 4B) for predicting pCR, with a sensitivity of 75.0%, specificity of 84.6%, PPV of 81.8%, NPV of 78.6%, and a diagnostic accuracy of 80.0%. The AUC between AFP response and radiographic response was not statistically different (0.798 vs 0.727, *P* = 0.568).

When the radiographic response was combined with the AFP response to serve as a predictor of pCR, we observed an AUC of 0.926 (95% CI: 0.785–1.0, Fig. 4C) in 25 patients who had positive AFP at baseline. The optimal cutoff value to predict pCR was 0.446, with a sensitivity of 91.7%, specificity of 84.6%, PPV of 84.6%, NPV of 91.7%, and a diagnostic accuracy of 88.0%. The AUC of this combination was marginally statistically significant, compared to the AUC of both radiographic response and AFP response (*P* = 0.067 and *P* = 0.051, respectively).

The probability for achieving a pCR can be calculated using the following formula: $\ln\frac{Prob}{1-Prob}=\left(4.010\times\mathrm{AFP}\;\mathrm{response}\right)+\left(5.170\times\mathrm{radiographic}\;\mathrm{response}\right)-5.304$; *Prob* in this formula stands for the probability of achieving a pCR. Among patients with positive AFP before initiation of systemic therapy, the AFP response was taken as 0 if the AFP was positive before surgery and 1 if the AFP was negative before surgery. The radiographic response was the percentage of reduction in the enhanced area in MRI.

The optimal cutoff value for predicting a pCR was 0.446 for this formula (i.e., $ln\frac{Prob}{1-Prob}\geq0.446$), which can only be used in patients with positive AFP before initiation of systemic therapy. In this case, fixing the AFP response allowed us to calculate the radiographic response. For patients with negative AFP before surgery (i.e., AFP response = 1), the reduction of tumor size needs to exceed 33.7% using the mRECIST measurement method to achieve a pCR. While for patients with positive AFP before surgery (i.e., AFP response = 0), the reduction of tumor size needs to exceed 111.2% using the mRECIST measurement method to achieve a pCR. However, in a real-world situation, the reduction of tumor size cannot exceed 100%, which means that patients with positive AFP both before initiation of systemic therapy and surgery (i.e., AFP response = 0) are more unlikely to achieve a pCR. In patients did not achieve AFP response, the pCR rate was 21.4% (3/14), which was significantly lower than the pCR rate (81.8% [9/11]) of patients achieved AFP response (*P* = 0.009, Table 2). Among patients with positive AFP before initiation of systemic therapy but negative AFP before surgery (i.e., AFP response = 1), the probability of achieving a pCR for those with MRR (i.e., a reduction of tumor size ≥ 80%) is ≥ 94.5%, which was validated as shown in Table 3.

**Table 3:** Relationship between combinations of AFP response and major radiographic response and pCR

AFP response^a^	-	-	+	+
Major radiographic response	-	+	-	+
Non-pCR	9 (100)	2 (40)	2 (33)	0 (0)
pCR	0 (0)	3 (60)	4 (67)	5 (100)

Data are presented as n (%)

^a^AFP response was defined as a change from a positive status at baseline to a negative status before resection. Of all 35 patients, 25 patients had positive AFP at baseline

*AFP* α-fetoprotein, *pCR* Pathologic complete response

## Discussion

To our knowledge, this is the first report demonstrating the association between radiographic response and pathological response to combined TKI/anti–PD-1 antibody therapy in patients with advanced HCC. Our results showed a clear relationship between the reduction of tumor size assessed using mRECIST measurement method (i.e., the percentage of reduction in tumor size of enhanced tumor area in MRI) and pCR. Furthermore, we also demonstrated that patients who achieved a pCR following systemic therapy before undergoing resection had a longer TTR versus those who did not achieve a pCR. This is comparable to previous studies showing better survival outcomes for patients with HCC who achieve a pCR to neoadjuvant locoregional therapy before undergoing liver transplant [14, 16].

Although many studies report pathological response based on fine needle biopsy, intratumoral heterogeneity may lead to false positive or false negative results with this method [26, 27]. Instead, surgical specimens obtained from resected tumor tissues after systemic treatment are the only way to accurately evaluate treatment response. Therefore, although this study includes a relatively small number of patients (*N* = 35), it is one of the largest studies to date examining conversion resection cases following a novel systemic therapy (TKI plus anti–PD-1). It is also the largest analysis comparing radiographic and pathological assessments of treatment response across multiple centers.

In alignment with previous studies, the present study showed that radiographic tumor response per mRECIST (CR, PR, SD, or PD) did not correlate with pathological response, which led to an underestimation of patients achieving a pCR. Even objective response, which includes cases classified as CR and PR, did not have a clear correlation with pCR. We believe this is because systemic therapy–induced tissue necrosis leads to inflammatory cell tissue infiltration and edema that can manifest as arterial phase enhancement and appear as viable tumor tissue.

We propose a definition of MRR—that is, the optimal cutoff value for predicting a pCR by radiographic imaging—to be a > 80% reduction in tumor size measured by the mRECIST measurement method, which helped to noninvasively and early identify patients who are most likely to benefit from conversion therapy strategy. Our ROC analysis showed that this threshold was associated with a 77.1% diagnostic accuracy for predicting pCR. For patients with HCC who had an AFP positive status before systemic treatment, the ability of radiographic response combined with AFP response for predicting pCR was even more accurate. The reason why the AUC for the combination of radiographic and AFP response (AUC = 0.926) is not significantly larger than that of the radiographic (AUC = 0.727) or AFP response (AUC = 0.798) alone may be attributed to an insufficient sample size when comparing two AUCs. Furthermore, our results showed that patients who achieved a pCR after systemic therapy had a longer TTR following resection versus those not achieving a pCR. We believe that our definition of MMR has high clinical value and should be validated through further studies.

Previous studies showed that radiographic CR per mRECIST and pCR were inconsistent [16, 28]. However, none of these studies explored the association between percentage reduction in tumor size and pCR. In contrast, our study investigated the association between radiographic response (i.e., percent reduction) and pathological assessment of tumor response in patients with advanced HCC who had received combined treatment with a TKI and an anti–PD-1 antibody without locoregional therapy before undergoing hepatectomy. Currently, the gold standard marker for assessing response to treatment is pCR, but this is an invasive method; thus, noninvasive methods for predicting pCR are required. This is particularly important, since we found a large difference in TTR between patients who achieved a pCR versus those who did not following surgical resection. An established noninvasive radiographic marker to predict pCR following systemic therapy in HCC would provide guidance on the prognosis and optimal timing of resection, as well as help identify when systemic treatment should be continued.

Our study had several limitations. First, the combination therapies used are not officially approved for use, and the treatment regimen was not unified as patients had different combinations of TKI plus an anti-PD-1 antibody. Second, the number of patients included was relatively small. In addition, all patients included in the study achieved a good treatment response and were able to undergo surgical resection, which represents a selection bias. Nevertheless, we believe our results represent an important preliminary step in the development of imaging criteria for evaluating patients undergoing conversion therapy with the intention of becoming a candidate for surgical resection. Third, our definition of MRR is only preliminary and requires further exploration in larger, prospective, and multicenter trials. Fourth, as other tumor makers, such as protein induced by vitamin K absence-II, alpha-fetoprotein-L3% and alpha-L-fucosidase, were not available to be assessed in all hospitals, this study did not investigate the relationship between other tumor markers alone or combined with radiographic evaluation and pathological tumor response, which could have further improved the predictive accuracy for pCR. Fifth, since the response of intrahepatic lesions to systemic therapy is not always the same as the response of extrahepatic lesions [10], the radiographic response of intrahepatic lesions cannot be used to predict the pathological response of extrahepatic lesions.

## Conclusions

In conclusion, patients with initially unresectable HCC who received combination therapy of a TKI with an anti–PD-1 antibody had a longer TTR after hepatectomy if they had achieved pCR after systemic therapy. Radiographic response alone or radiographic response combined with AFP response can predict pCR in tumors of these patients.

## Data Availability

The datasets used and/or analyzed during the current study are available from the corresponding author on reasonable request.

## Abbreviations

pCR: Pathologic complete response
HCC: Hepatocellular carcinoma
TKI: Tyrosine kinase inhibitor
PD-1: Programmed death 1
AUC: Area under the receiver operating characteristic curve
ORR: Objective response rate
mRECIST: Modified response evaluation criteria in solid tumors
AFP: α-fetoprotein
HBV: Hepatitis B virus
HBsAg: Hepatitis B surface antigen
MRI: Magnetic resonance imaging
CR: Complete response
PR: Partial response
RVT: Residual viable tumor
SD: Stable disease
PD: Progressive disease
TTR: Time to recurrence
ROC: Receiver operating characteristics
